# Conformational Sampling and Binding Site Assessment of Suppression of Tumorigenicity 2 Ectodomain

**DOI:** 10.1371/journal.pone.0146522

**Published:** 2016-01-06

**Authors:** Chao-Yie Yang, James Delproposto, Krishnapriya Chinnaswamy, William Clay Brown, Shuying Wang, Jeanne A. Stuckey, Xinquan Wang

**Affiliations:** 1 Department of Internal Medicine, Hematology and Oncology Division, University of Michigan, Ann Arbor, Michigan, 48109, United States of America; 2 Life Sciences Institute, University of Michigan, Ann Arbor, Michigan 48109, United States of America; 3 Department of Microbiology and Immunology, National Cheng Kung University Medical College, Tainan 701, Taiwan; and Center of Infectious Disease and Signaling Research, National Cheng Kung University, Tainan 701, Taiwan; 4 Biological Chemistry, University of Michigan, Ann Arbor, Michigan 48109, United States of America; 5 Ministry of Education Key Laboratory of Protein Science, Center for Structural Biology, School of Life Sciences, Tsinghua University, Beijing 100084, China; Instituto de Tecnologica Química e Biológica, UNL, PORTUGAL

## Abstract

Suppression of Tumorigenicity 2 (ST2), a member of the interleukin-1 receptor (IL-1R) family, activates type 2 immune responses to pathogens and tissue damage via binding to IL-33. Dysregulated responses contribute to asthma, graft-versus-host and autoinflammatory diseases and disorders. To study ST2 structure for inhibitor development, we performed the principal component (PC) analysis on the crystal structures of IL1-1R1, IL1-1R2, ST2 and the refined ST2 ectodomain (ST2^ECD^) models, constructed from previously reported small-angle X-ray scattering data. The analysis facilitates mapping of the ST2^ECD^ conformations to PC subspace for characterizing structural changes. Extensive coverage of ST2^ECD^ conformations was then obtained using the accelerated molecular dynamics simulations started with the IL-33 bound ST2^ECD^ structure as instructed by their projected locations on the PC subspace. Cluster analysis of all conformations further determined representative conformations of ST2^ECD^ ensemble in solution. Alignment of the representative conformations with the ST2/IL-33 structure showed that the D3 domain of ST2^ECD^ (containing D1-D3 domains) in most conformations exhibits no clashes with IL-33 in the crystal structure. Our experimental binding data informed that the D1-D2 domain of ST2^ECD^ contributes predominantly to the interaction between ST2^ECD^ and IL-33 underscoring the importance of the D1-D2 domain in binding. Computational binding site assessment revealed one third of the total detected binding sites in the representative conformations may be suitable for binding to potent small molecules. Locations of these sites include the D1-D2 domain ST2^ECD^ and modulation sites conformed to ST2^ECD^ conformations. Our study provides structural models and analyses of ST2^ECD^ that could be useful for inhibitor discovery.

## Introduction

Suppression of Tumorigenicity 2 (ST2), is a member of the interleukin-1 receptor (IL-1R) family[1]. Two ST2 isoforms, i.e. the membrane-bound ST2[2] and the alternatively spliced soluble ST2 (sST2)[3,4], have been well studied. Binding of the membrane-bound ST2 with IL-33 (the only known ligand) leads to their association with IL-1RAcP; thereby, activating the downstream MyD88, p38 and NF-κB regulated genes[5] known as the ST2/IL-33 axis. Soluble ST2 circulates in blood and contains the same ectodomain as ST2 that binds with IL-33. Activation of ST2/IL-33 axis on T helper 2 (T_H_2) cells results in the release of T_H_2 associated cytokines including IL-4, IL-5, IL-13 and IL-9[6]. The T_H_2 associated cytokines cause accumulation of eosinophil and promote T_H_2 cells polarization[7]. While ST2/IL-33 signaling is important for T_H_2 immune response to tissue repair and helminth infection[8], it has been associated with immunity-related diseases including asthma, allergy, cardiovascular disease, central nervous system disease, pain and arthritis[9]. For example, an excessive T_H_2 cells immune response from ST2/IL-33 signaling[10] was found in some type of asthma patients[11]. In another case, Miller et. al. [12] showed exogenous administration of IL-33 shifts T_H_1 to T_H_2 response via the ST2/IL-33 axis which alleviated inflammation in a mouse model of atherosclerosis. In allogeneic hematopoietic stem cell transplantation, elevated level of sST2 in plasma is a predicted risk factor to the graft-versus-host disease (GVHD) mortality[13]. For GVHD, sST2 (as a decoy receptor) attenuates the IL-33 pool available for the membrane-bound ST2 to dampen T_H_2 response. The resulting polarization towards the T_H_1 population contributes to severe gastrointestinal tract damages in acute GVHD[14] and mortality.

Because ST2/IL-33 signaling is associated with multiple pathological diseases, the pathway has emerged as a novel therapeutic target[9,15]. Direct intervention of ST2 and IL-33 binding by inhibitors can prevent downstream signaling activation of T_H_2 response. Alternatively, the molecules can alter the pool of IL-33 in plasma by releasing IL-33 from sST2 to bind with the membrane-bound ST2 in different tissues and modulate T_H_2 response. Currently, no small molecules or protein-based therapeutics to inhibit IL-33 binding with ST2 have been reported or approved, likely due to the late discovery of IL-33[6]. In comparison, many antibody-based therapies to target IL-1R1 (a founding member of IL-1R family)/IL-1β signaling pathway have been reported[16]. They include FDA-approved Anakinra (an analog of the IL-1R antagonist, IL-1Ra)[17] and Canakinumab (an anti-IL-1β monoclonal antibody)[18] for treating rheumatoid arthritis and cryopyrin-associated periodic syndromes. Therapeutic options to target the ST2/IL-33 axis can be anticipated in near future.

In inhibitor development, target structures provide insights to identify important interactions with their ligands and rationales to design inhibitors[19,20]. ST2 and sST2 both share the same ectodomain (sequence: 1–323, denoted as ST2^ECD^) which consists of three immunoglobulin-like domains called D1, D2, and D3. Only one structure of the ST2/IL-33 complex is available which shows ST2^ECD^ interacts with IL-33 using all D1-D3 domains[21]. We analyzed the structure and found the contact surface area between ST2 and IL-33 is 3631.5 Å^2^ (typical for protein-protein interaction[22] but challenging for small molecule inhibitor development). Although site-directed mutagenesis experiments indicated that two residues (D149, E165) of IL-33 at the interface contribute significantly to ST2/IL-33 interaction[21,23], yet it remains to be determined if the site interacting with D149 and E165 can bind with potent small molecules. Besides the ST2/IL-33 crystal structure, previous studies[21,23] also suggested that the D1-D2 domain of ST2 is rigid whereas the D3 domain is highly mobile. This was supported by that the D3 domain of the ST2^ECD^ models derived from small-angle X-ray scattering (SAXS) data by Liu et. al. [21] displays three different orientations but not the D1-D2 domain. We examined these ST2^ECD^ models and found an unfavorable interaction between a loop and the D3 domain which prohibits follow-up investigation. While these data elucidate structural information of ST2, feasibility of binding between ST2 and small molecule ligands has not been explored. Recently, we reported that the flexible inter-domain motion in IL-1R type 1 (IL-1R1) ectodomain is mainly between the D2 and the D3 domain of IL-1R1[24]. Our previous study implicated a full structural characterization of ST2^ECD^ ensemble conformations will be necessary to identify and assess potential small molecule binding sites of ST2^ECD^.

In this work, we generated conformational ensemble of ST2^ECD^ using the efficient accelerated molecular dynamics (aMD) conformational sampling method starting with the well-defined IL-33-bound ST2^ECD^ structure. ST2^ECD^ conformations were analyzed based on principal component analysis (PCA) of the IL-1R1/2, ST2 crystal structures and the refined ligand-free ST2^ECD^ models using previously collected small-angle X-ray scattering (SAXS) data. Cluster analysis of the conformations was then carried out to select representative ST2^ECD^ models. Comparison of the representative conformations with ST2/IL-33 and the experimental binding data both suggested that the D1-D2 domain of ST2 plays a dominant role to interact with IL-33. Computational binding site assessment of the representative conformations further determined locations in ST2^ECD^ that may interact with small molecules. Finally, we discussed the strategies to discover small molecule inhibitors targeting ST2^ECD^.

## Materials and Methods

### Crystal structures used in this study

All protein structures labeled with PDBIDs were obtained from the Protein Data Bank[25]. They included the protein complex structures of ST2/IL-33 (PDBID: 4KC3[21]), IL-1R1/IL-1β (PDBID: 1ITB[26]), IL-1R1/IL-1β/IL-1RAcP (PDBID: 4DEP[27]), IL-1R1/IL-1Ra (PDBID:1IRA[28]), IL-1R1/EBI-005 (PDBID: 4GAF[29]), IL-1R1/AF10847 (PDBID: 1G0Y[30]), and IL-1R2/IL-1β/IL-1RAcP (PDBID: 3O4O[31]). Among them, EBI-005 is an IL-1β chimera and binds more potently to IL-1R1 than IL-1ß[29]. The ectodomains of IL-1R1/2 and ST2 were extracted from each structure for analysis. In the ST2 ectodomain structure, residues Q52-K55, E224-A231, Q272-N278 were unresolved. We modeled these missing segments using default parameters in the MOE[32] program. All proteins shown in the figures were prepared by UCSF Chimera program[33].

### Generation of ST2^ECD^ models using AllosMod-FoXS

The ST2 crystal structure corresponding to K19-N323 was used as the template structure to construct the SAXS derived ST2^ECD^ models. Missing residues in the crystal structure and the C-terminal Histag (six Histidine residues) were built using the MOE[32] program. All ST2^ECD^ models were generated by the AllosMod-FoXS web server[34] by fitting to the SAXS data. The most probable models setting was selected in the model calculations. Single state and ensemble models of up to 4 states determined by MultiFoXS and Minimal Ensemble Search (denoted as MES) algorithms were saved for analysis. The χ values of fitting to the SAXS profile in MultiFoXS were 1.96, 1.87, 1.78, 1.76 and those in MES were 1.58, 1.43, 1.43, 1.43 in the one-, two-, three-, and four-state models respectively.

### MD simulation setup

ST2^ECD^ (sequence: K19-N323) from the crystal structure was used in the MD simulations. Three N-Acetyl-D-Glucosamine bonded to N95, N140, N151 were removed whereas five pairs of cysteine residues forming disulfide bonds were bonded during simulations. To prepare protein structures for simulations, we have used the MOE program[32] to assign the protonation state of ionizable groups in ST2^ECD^ under the pH 7.4 physiological condition. The Amber 99SB force field parameters[35] were used for the amino acids and the TIP3P[36] model for water molecules. The physiological salt concentration at 150 mM of NaCl was adopted in the simulation. The protein was embedded in an octahedron water box in which the distance between any protein atom to the edge of the box was set at 15 Å. The overall charge of the system was set neutral. The GPU modification to PMEMD[37] from Amber (version 12)[38] was used in all MD simulations.

Preparation of the system for production run used the following procedures. A 3000-step minimization (steps 1–1000 using conjugated gradient followed by 2000 steps steepest decent) was first carried out. After minimization, a 50 ps constant volume and constant temperature (NVT) simulation was performed to raise the temperature of the system to 298K while constraining all heavy atoms with a 5 kcal/mol/Å^2^ force constant with reference to the starting conformation. Using a 1 kcal/mol/Å^2^ force constant to constrain all heavy atoms, the system underwent eight additional simulating annealing type of temperature changes under isobaric (P = 1 atm) conditions. The procedures included series of 50 ps runs by changing the temperatures from 298K to 350K, 350K to 400K, 400K to 450K, 450K to 500K, 500K to 550K respectively. Then, a 100 ps run was done at T = 550K followed by 50 ps runs with the temperature decreasing from 550K to 425K and 425K to 298K. Finally, a one ns pre-equilibration run with T = 298K without any constraint to protein atoms was performed in the isobaric isothermal environment (NPT, T = 298K and P = 1 atm) before the production run. The SHAKE[39] algorithm was used to fix bonds involving hydrogen atoms. The Particle mesh Ewald method[40] was used and the non-bonded cutoff distance was set at 10 Å. The time step was 2 fs, and neighboring pairs list was updated every 20 steps.

For performing the accelerated MD (aMD) simulations, the first 2 ns production run based on the conventional MD (no modification of the potential energy function) was used to determine the average values of the potential energy for the total system (V_total_) and the dihedral angle energy of the proteins (V_dih_). The threshold potential energy was defined as V_total_ + N_atoms_/5 (N_atoms_: total number of atoms in the system). Four threshold potential energies for the dihedral angle motion were used called AMD1, AMD2, AMD3 and AMD4 which were equal to Vdih + (number of residues) × 3.5, 4.2, 4.9, and 5.6 respectively. These parameters were taken as suggested by previous work[41]. A total simulation time of 120 ns was collected from 30 ns runs of aMD using AMD1-AMD4 parameters.

### Principal component analysis and mean shift cluster analysis

The crystal structures of IL-1R1, IL-1R2, ST2 ectodomains used in the PCA were extracted from the complex structures of IL-1R1 with IL-1β (PDBID: 1ITB, chain B), IL-1Ra (PDBID: 1IRA, chain Y), AF10847 (PDBID: 1G0Y, chain R), EBI-005 (PDBID: 4GAF, chain B), IL-1β and IL-1RAcP (PDBID: 4DEP, chains B and E), IL-1R2 with IL-1β and L-1RAcP (PPDBID: 3O4O, chain C), and ST2 with IL-33 (PDBID: 4KC3, chain B). ST2 models included in the PCA were the 1- and 3-state models determined from the MultiFoXS and MES algorithms in this work. A total of 15 structures were analyzed using the PCA implemented in the Bio3D version 2.0[42]. Detailed procedures of the PCA can be found at the Bio3D website (http://thegrantlab.org/bio3d/) and our previous report[24].

After the principal components (PCs) were determined, snapshots of ST2^ECD^ conformations obtained from aMD simulations were projected to the PC subspace. The coefficients of the first three components for each conformation were used to depict a point in the three dimensional PC subspace in all figures. Based on the PCA, we found the first seven principal components were sufficient to characterize 99% of structural variations in the 15 structures. The first seven PCs were then used in the mean shift cluster analysis provided in the Scikit-Learn program[43]. Mean shift cluster analysis is a nonparametric clustering method[44–46]. Unlike k-mean clustering method, the mean shift clustering requires no prior knowledge of the number of clusters in the multidimensional data. The algorithm classifies data points by moving the data point via the density gradient defined by a kernel density estimator function with an assigned bandwidth until it reaches the maximum of a local density distribution called mode in pattern recognition. In our application here, each mode is the center of a cluster group. To perform the mean shift cluster analysis, snapshots of ST2^ECD^ conformations at every 10 ps from the aMD simulations were included. The quantile value was varied from 0.01 to 0.20 in the bandwidth estimation to analyze the stability of the numbers of cluster groups. Conformations closest to the centroid of each cluster group were selected to represent each cluster and denoted as representative conformations.

### Sitemap analysis

We used Sitemap program[47] to perform small molecule binding site analysis on the protein conformations. The Sitemap program developed by Halgren[47] is an in silico method that detects the cavities in proteins and calculates their sizes, physicochemical properties (hydrophobic surface, hydrogen bond acceptors, donors) probed by chemical atoms, solvent exposure that are important to analyze protein-ligand interaction. These properties were weighted to derive scoring functions (including Dscore) for evaluating the binding sites by training and testing to a dataset with known protein-ligand crystal structures and associated binding affinities. The correlation between the calculated Dscore values and the experimental binding affinities of known ligands to their target proteins gives a measure to predict the likelihood of novel protein binding sites to bind with potent small molecules and the druggability of proteins.

Representative conformations determined by the cluster analysis were used in the Sitemap program[47] to identify potential small molecule binding sites. Each conformation was first processed in the Glide program[47] from the Maestro 9.7 program suite (Release 2014–1)[47] followed by the Sitemap analysis using default parameters. Up to five binding sites were saved for binding sites analysis. Druggability index of each binding site was based on the Dscore value in the Sitemap program. The volume of each binding site was calculated by the Sitemap program.

### Protein expression and purification

Oligos for PCR construction were designed using Clone-manager (Scientific and Educational Software). IL-33 (residues 112–270) was cloned into an N-terminal His6-TEV expression vector, which was then transformed into Rosetta2^™^ (DE3) cells. Cultures were grown at 37°C to an OD_600_ of 1.2 in Terrific Broth, induced with 0.4 mM IPTG and expressed overnight at 20°C. Cells were lysed by sonication in 10 mM HEPES, pH 7.5, 150 mM NaCl, 0.1% β-mercaptoethanol with protease inhibitors and cellular debris pelleted by centrifugation. The resulting supernatant was incubated with Ni-NTA resin (Qiagen) equilibrated with 10 mM HEPES, pH 7.5, 150 mM NaCl, 10 mM imidazole for 1 hr at 4°C and eluted with same buffer containing 300 mM imidazole. Tag removal occurred by dialyzing the eluate against 10 mM HEPES, pH 7.5, 150 mM NaCl and 1 mM DTT overnight at 4°C in the presence of TEV protease and incubating the protein with an additional Ni-NTA column. The flow through containing the protein was concentrated and loaded on to a Superdex 75 column (GE Heathcare) equilibrated with 10 mM HEPES, pH 7.5, 150 mM NaCl. The purified protein was stored at -80°C in the same buffer containing 10% glycerol.

ST2 constructs 19–206 and 19–323 were cloned into pH-HBM-7. This vector has a honeybee melittin signal sequence followed by a His tag and a TEV protease cleavage site[48]. Expression trials indicated that High-five cells infected for 72 hours displayed the best yield of secreted protein. The conditioned media from two 1 L infection cultures for each construct was pooled for purification. All ST2 constructs were purified with the protocol described below. Media containing the expressed ST2 constructs were incubated with Ni-NTA resin (Roche) pre-washed with 10 mM HEPES, pH 7.2 and 150 mM NaCl (denoted Buffer A) for 4 hours at 4°C using a magnetic stirrer. Bound media was collected in a column and washed with Buffer A. The ST2 proteins eluted with Buffer A contained 300 mM imidazole and were dialyzed overnight at 4°C against Buffer A with TEV protease. The protein solution was then reapplied to a Ni-NTA column to remove the tag, then concentrated and applied to a Superdex 200 column (GE Healthcare) equilibrated with Buffer A.

For Bio-layer interferometry, the purified ST2 proteins and IL-33 were biotinylated by diluting the protein to 0.5 mg/mL in Buffer A, adding an equimolar ratio of Biotin-PEO4 linker (Pierce PN 21329) and incubating on ice for 2 hours. Unbound linker was removed via dialysis against 50mM sodium phosphate and 300 mM NaCl, pH 7.5.

### Bio-layer Interferometry

BLI experiments were performed using an OctetRED96 instrument from ForteBio. All experiments were conducted at 25°C using 10 mM HEPES, pH 7.5, 150 mM NaCl, 0.005% Tween 20, 100 μM EDTA as the assay buffer. Assays were conducted in Greiner 96 well black flat-bottom microplates and were continuously shaken at 1000 RPM before measurements. Biotinylated ST2 was first loaded onto the Streptavidin (SA) sensors for 10 minutes at 20 μg/mL to achieve complete loading saturation. Complete loading reduced nonspecific binding which occurred on unloaded sensors. Serial dilutions of IL-33 were prepared on the Greiner plates. For ST2^ECD^, new sensors were used for every IL-33 concentration due to slow dissociation. For ST2^D1-D2^, dissociation was complete in a short amount of time and sensors were reused between different IL-33 concentrations. In each sensorgram measurement, sensors loaded with ST2 were first equilibrated in the reference buffer for 10 minutes, then associated with IL-33 for 1 minute and dissociated for 1 minute for ST2^D1-D2^ whereas the association and dissociation times for ST2^ECD^ were 15 minutes, and 15 minutes up to 2 hours, respectively. 50 μg/mL of biotinylated IL-33 was loaded onto sensors to serve as a negative-control parallel reference sensor. Raw kinetic data collected were processed using ForteBio data analysis software 7.1 and then analyzed in Prism 6.0 (GraphPad Software, La Jolla, California USA). Analysis of the data based on a 1:1 binding model gave k_on_ and k_off_ values which yielded the calculated K_D_ values.

## Results and Discussions

### Refined models of ST2^ECD^ based on the SAXS data

A ST2 homology model was used previously[21] to derive three structural models by fitting to the SAXS data of ST2^ECD^ employing the minimum ensemble search (MES) algorithm in BILBOMD[49]. Examination of these models showed that a flexible loop (Q272 to N278, unresolved in the ST2/IL-33 crystal structure) penetrated into the D3 domain. Further, the distances between C238 and C282 in all three models were 21 Å which are incompatible with a disulfide bond distance observed in the crystal structure. The large distance separation between C238 and C282 originated from the ST2 homology model template and the lack of distance constraint between C238 and C282 in the model construction.

**Fig 1:**
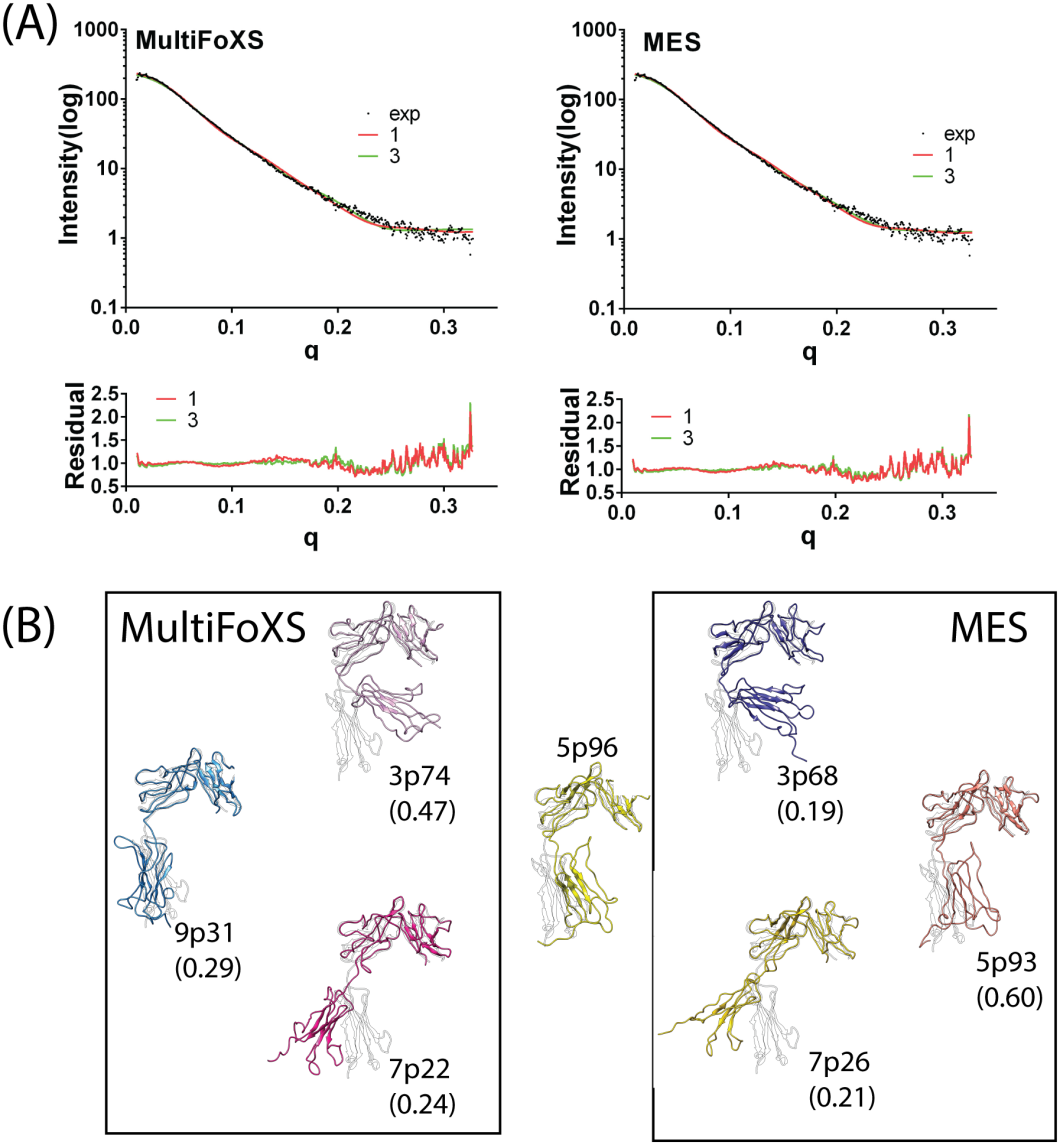
Models of ST2 fitted to the SAXS data using MultiFoXS and MES algorithms. (A) Fitting curves and residual plots of the 1-state and 3-state models based on MultiFoXS and MES. (B) ST2^ECD^ models determined by MultiFoXS and MES. 5p96 is the 1-state model. Each model was aligned with the IL-33 bound ST2 structure (grey). The number in the parentheses corresponds to the weight of each conformation in the 3-state model.

Here, we used the well-resolved ST2^ECD^ template structure extracted from the ST2^ECD^/IL-33 crystal structure and the web-based program, AllosMod-FoXS[34], to build the ST2^ECD^ models by fitting to the same SAXS data. AllosMod-FoXS provides a single dominant ST2^ECD^ conformation and also constructs ensemble models based on either multi-state model or minimum ensemble search algorithm (denoted as MultiFoXS and MES respectively). Both independently developed algorithms were considered in our analysis to derive dominant ST2^ECD^ conformations in the ensemble that fit with the SAXS data. The flexibility of ST2^ECD^ in solution has been discussed previously. The Porod-Debye plot showed the loss of plateau (S1 Fig) and the Porod exponent is 3.5 at the linear region further suggested some degrees of flexibility in ST2^ECD^[50]. Because the χ values between 3- and 4- state models improved only marginally for MultiFoXS and MES, we adopted the 3-state models as the ensemble model for ST2^ECD^ to avoid data overfitting in the 4-state models. The χ values of the 3-state model determined by MultiFoXS and MES are similar (1.78 versus 1.43). The fitting curves and residual values between 1- and 3-state models from MultiFoXS and MES are provided in Fig 1A.

The SAXS-derived 1- and 3-state models of ST2^ECD^ determined by MultiFoXS and MES are shown in Fig 1B. 5p96 represents the dominant 1-state model fit to the SAXS data. The 3-state models selected by MultiFoXS are 3p74(47%), 9p31(29%), and 7p22(24%) whereas those by MEM are 3p68(19%), 5p93(60%), and 7p26(21%). Visual inspection of the models suggested that similar conformations were found in the models determined by MultiFoXS and MES. For examples, 3p74 and 3p68, 7p22 and 7p26 are similar, and 9p31 and 5p93 exhibit similar conformations to each other and to 5p96. More quantitative comparison will be discussed in the following section. Convergence of the backbone conformations derived from the SAXS data by two algorithms gave confidence that the models are representative ST2^ECD^ conformations in solution.

Most models also showed reasonably close distances between matching pairs of 10 cysteine residues forming disulfide bonds. Exceptions were found in three models (9pm31, 3pm68, 5pm93) in which distances between C111 and C151 were 8.1, 8.1 and 11.2 Å. C111 and C151 are both in loop regions and have largest solvent accessible surface areas than the other eight cysteine residues according to the ST2 crystal structure. The bonding between C111 and C151 is expected to constrain the D1 and D2 domains in the dynamical motion of ST2 but not the overall backbone conformation of the three models. Alignment of these models with the ST2 crystal structure (Fig 1B) demonstrated that the flexibility of ST2^ECD^ observed in solution can be attributed to the orientational motion of the D3 domain with respect to the D1-D2 domain mediated by a loop (sequence: K203–S209) between the D2 and D3 domains similar to the IL-1R1 ectodomain[24]. Most models also resemble three previous models[21] except 7p22 and 7p26 in which the D3 domain swings further away from the D1-D2 domain.

### Characterization of ST2^ECD^ conformations based on the principal component analysis

Previous studies have shown PCA as an informative tool to characterize protein conformational changes by analyzing experimental structures[51] and conformations obtained from MD simulations[52]. To analyze the ST2^ECD^ conformations and their changes in the MD simulations, we performed the principal component analysis (PCA)[53] by combining crystal structures and the SAXS derived models. Only one ST2^ECD^/IL-33 structure (PDBID: 4KC3) is currently available; however, IL-1R1, IL-1R2 and ST2 are in the same IL-1R family. The sequence identities of the ectodomains between ST2 and IL-1R1/2 are 18% and all contain the D1-D3 domains. The crystal structures of IL-1R1, IL-1R2, ST2 included in the PCA were described in the Materials and Methods. Although SAXS derived models cannot provide accurate atomic details of the ST2^ECD^ conformations, the PCA requires only the Calpha positions of the proteins which are characterized by the folds and shapes of the SAXS models[54]. Therefore, we included the SAXS derived models to supplement limited crystal structures, and to provide the ligand-free ST2^ECD^ conformations not observed in the ligand-bound crystal structures. The SAXS-derived ST2^ECD^ models included in the PCA are the 1- and 3-state models determined by MultiFoXS and MES. ST2^ECD^ conformations projected to the first three principal components (PC_123_) are displayed in all figures despite that all PCs were included in the analysis.

**Fig 2:**
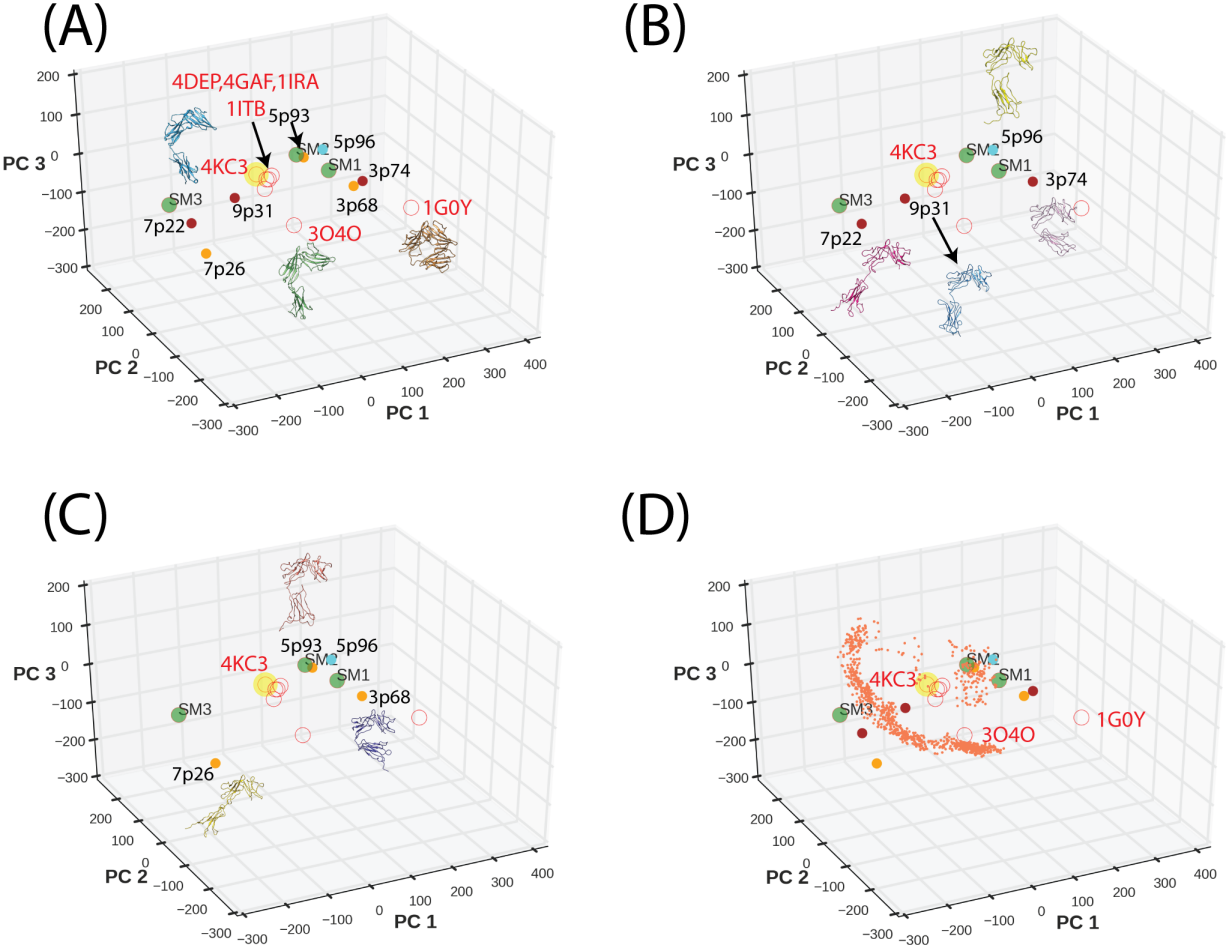
Projection of IL-1R1/2, ST2 crystal structures and ST2^ECD^ models to the first three principal components subspace. (A) Projected locations of the crystal structures and the SAXS-derived ST2^ECD^ models are shown in open and filled circles respectively. Brown circles are from MultiFoXS (B) and orange circles are from MES (C). PDBIDs of the crystal structures and the ST2^ECD^ models are labeled close to the symbols. The yellow and green filled circles denote the IL-33 bound ST2^ECD^ structure and three previously reported ST2^ECD^ models respectively. (D) Projection of the ST2^ECD^ conformations obtained from 22 ns of MD simulations.

In Fig 2A, we show that conformations of IL-1R1/2 and ST2 bound to IL-1β (PDBIDs: 1ITB, 4GAF) or IL-33 (PDBID: 4KC3) are mapped to the same region and the presence of IL-1RAcP did not influence the IL-1R1 conformation bound with IL-1β (PDBID: 4DEP). For the antagonist IL-1Ra-bound IL-1R1 (PDBID: 1IRA), the D3 domain oriented slightly different from that in the IL-1β-bound IL-1R1 and was mapped to a nearby location. In comparison, the IL-1R2 conformation (bound with IL-1RAcP and IL-1β (PDBID: 3O4O) was mapped further away from both ST2 and IL-1R1 along the PC2 degree of freedom. Our structural alignment indicated the D3 domain of IL-1R2 deviates more from that of IL-1R1. It is unclear whether the difference is associated with the IL-1RAcP binding because no other IL-1R2 structure is available. For the AF10847-bound IL-1R1 conformation (PDBID: 1G0Y), the D1 and D3 domains make close contact and its conformation was mapped to another location involving changes in PC1 and PC2 degrees of freedom.

The conformations and the projected locations of SAXS models determined by MultiFoXS and MES are shown in Fig 2B and 2C. Their locations differ from the known crystal structures and cover a wider range of PC subspaces. Based on the proximity in location, we identified similar conformations in 7p22/7p26, 3p74/3p68 and 5p93/5p96. Although three previous models were not included in the PCA, projection of their conformations to the PC subspace (green circles) indicated that SM1 and SM2 are close to 5p93 and 3p74. This suggests that SM1 and SM2 captured major conformational shapes because 5p93 and 3p74 have the largest weights in the 3-state models. We next projected the ST2^ECD^ conformations collected from 22 ns of MD simulation to the PC subspace in Fig 2D (orange circles). The data showed that the IL-33-bound ST2^ECD^ quickly relaxed from the initial conformation and underwent circular motion mostly along the PC2 degree of freedom. Fig 2 also demonstrated the complementarity between the crystal structures and the SAXS-derived ST2^ECD^ models to characterize ST2 ^ECD^ conformations.

### Conformational sampling of ST2^ECD^ using the accelerated MD method

**Fig 3:**
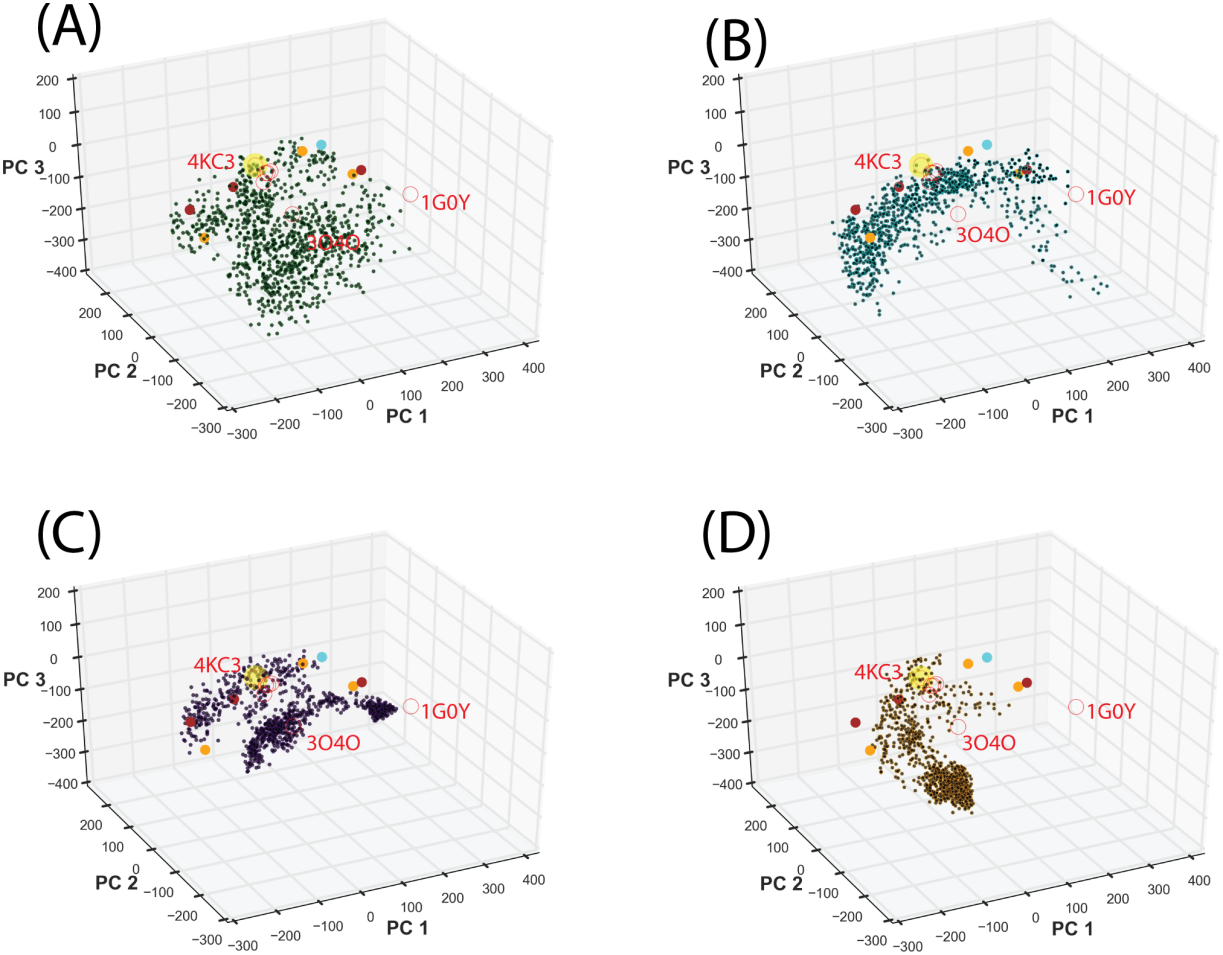
Projection of ST2^ECD^ conformations obtained from a total of 80 ns aMD simulations to the PC subspace. Projections of ST2^ECD^ conformations obtained from the aMD simulations using (A) AMD1, (B) AMD2, (C) AMD3, (D) AMD4 parameters. Yellow, red and filled circles represent the projected locations of ST2/IL-33, IL-1R1/2 crystal structures and the SAXS-derived models respectively.

Fig 2D demonstrated an example of limited conformational sampling using conventional MD simulations. To overcome local conformational sampling and access higher energy states compatible with ligand binding[55], we performed the accelerated MD (aMD) sampling[41] starting with the IL-33 bound ST2^ECD^ crystal structure. We have previously shown that aMD allows more efficient conformational sampling of IL-1R1 ectodomain than the conventional MD simulations for the same length of simulation time[24]. This was attributed to the adjustment of the potential energy function which allows the protein to cross energy barriers during simulations. Here, we used four sets of parameters in the aMD simulations, denoted by AMD1-4, to progressively produce shallower energy wells and access conformations separated by higher energy barriers in the energy landscape. Fig 3A shows that the AMD1 parameters allowed efficient and diverse conformational sampling when compared with those obtained from conventional MD simulations (cf. Fig 2D). Although parameters based on AMD2-4 provided conformational sampling of ST2^ECD^ in different PC subspaces, yet they are more localized. Combining ST2^ECD^ conformations from all 4 sets of parameters indicated that they provide broad coverage of PC subspace guided by the locations of the crystal structures and the SAXS-derived models. While the crystal structures cover some of the ligand-bound conformations, the SAXS-derived models represent the ligand-free ST2^ECD^ conformations in solution.

**Fig 4:**
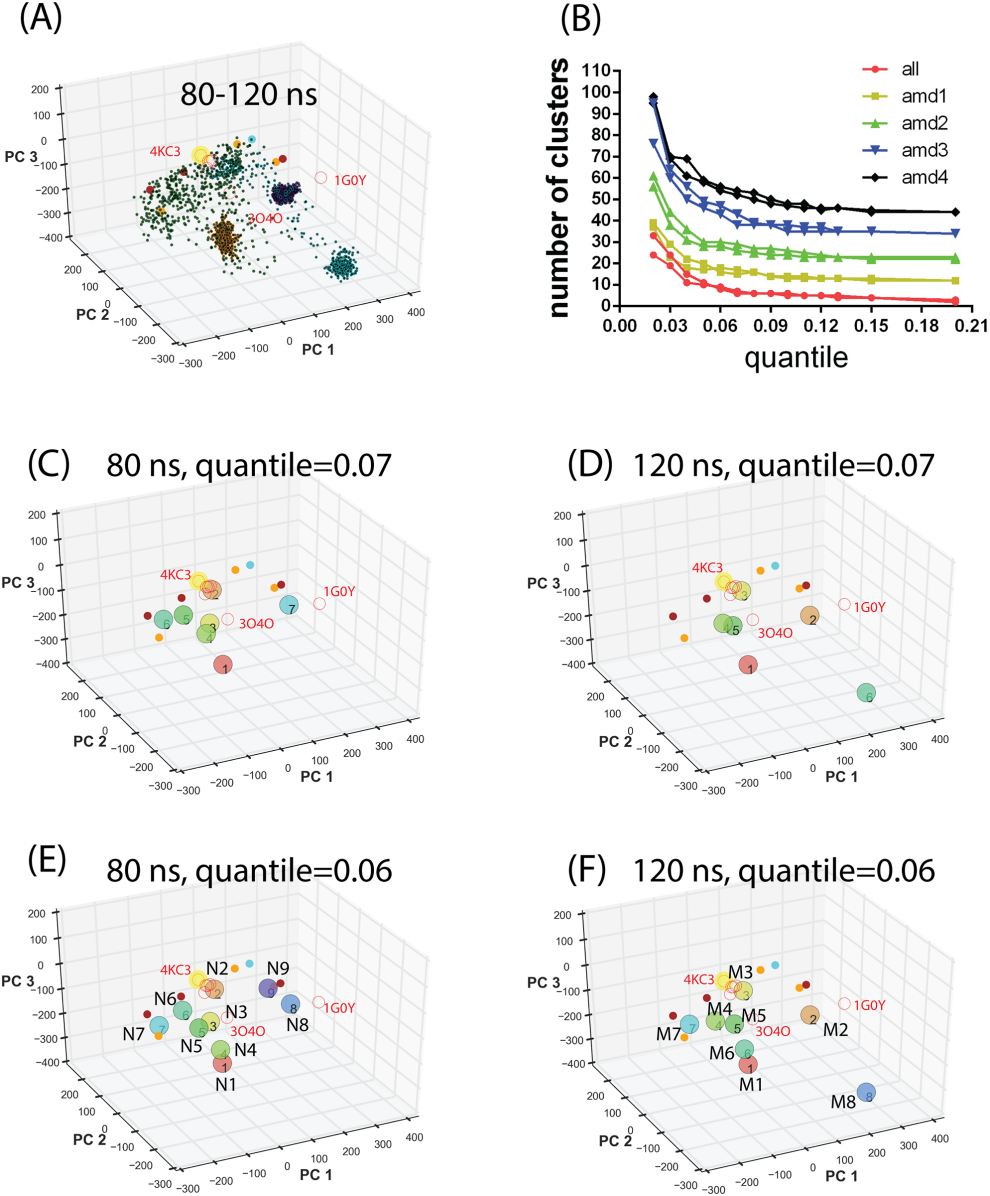
Projection of ST2^ECD^ conformations obtained from aMD simulations and the mean shift cluster analysis. (A) Projection of conformations corresponding to 20–30 ns of each aMD simulations colored according to different sets of parameters (see Fig 3). (B) Stability analysis of the number of cluster groups determined by the mean shift cluster analysis. (C-F) Centroids of cluster groups determined by the mean shift clustering using conformations from a total of 80 or 120 ns aMD simulations and the quantile values of 0.06 and 0.07. Yellow circles, red circles and filled circles denote the projected locations of ST2/IL-33, IL-1R1/2 crystal structures and the SAXS-derived models. The centroids of cluster groups 1–9 and 1–8 in (E) and (F) are relabeled as **N1**-**N9** and **M1**-**M8** respectively.

Each aMD simulation was extended by another 10 ns to determine the convergence of the sampling. Projections of the conformations from the extended simulations, shown in Fig 4A, indicated that the sampling using the AMD1 parameters remains diverse in the PC subspace while the conformations sampled using the AMD2-4 parameters were trapped to three separate localized regions. This suggested that conformations collected from a total of 80 ns simulations using AMD1-4 parameters covered the majority of the PC subspace and the extended 40 ns simulations gave only minor additional coverage of the PC subspace. To visualize the diverse ST2^ECD^ conformations obtained from the aMD sampling, we performed the mean shift cluster analysis to group similar conformations in the PC subspace into the same cluster. Each cluster group in the conformational ensemble of ST2^ECD^ is represented by the conformations corresponding to the centroid of the cluster.

In the mean shift cluster analysis, the number of data points and the quantile value in the bandwidth estimation can dictate the number of cluster groups. To assess the effects of both parameters on the number of cluster groups, we included 4000 and 6000 conformations taken from a total of 80 and 120 ns of aMD simulations and varied the quantile value from 0.2 to 0.01. The results shown in Fig 4B indicated that setting the quantile value between 0.12 and 0.06 leads to relatively stable number of clusters reflecting the stability of data classification in the cluster analysis[44]. When the quantile value is lower than 0.04, the number of clusters starts to increase substantially. The number of conformations included in the analysis (80 ns versus 120 ns) made small differences to the number of clusters determined. We further compared the centroids of the clusters between 80 and 120 ns using the quantile value = 0.07 and 0.06 in Fig 4C–4F. In Fig 4C and 4E, locations of cluster 2, 3, 5, 6, and 7 were not changed between the two quantile values. Similar trend was found in Fig 4D and 4F. When the quantile value is 0.06, we found higher resolution of clusters in the regions covered by clusters 1, 2, 3 5 and 6 in Fig 4C or clusters 1, 4 and 5 in Fig 4D. The change of the **M2** location and emergence of **M8** between Fig 4F and 4E can be attributed to increased conformational populations in the PC subspaces from the extended sampling simulations as shown in Fig 4A. Comparison between Fig 4E and 4F indicated variations in the locations of **N8**, **N9, M2,** and **M8** but not other cluster groups. This reflects that increased population of certain localized states in the extended aMD simulations may bias the mean shift cluster analysis to select these stationary states. To retain the diversity of representative conformations, we included **N1**-**N9** and **M1**-**M8** in the subsequent analysis.

To determine the relation between these representative conformations with the SAXS models, we calculated the Euclidean distances between them as listed in Table 1. Because these conformations are projected to the PC subspace, the Euclidean distance is not directly related to distance in angstrom for interpretation. However, if we use the distance of less than 100 a.u. in Table 1 to infer the closeness between conformations, we found that 9 of 17 representation conformations are close to some of the SAXS derived models (shown in bold italic fonts in Table 1). Based on this analysis, the ensemble of conformations obtained from aMD recapitulates the states represented by most of the SAXS derived models except 3p74. Backbone RMSD values of the D1-D3 domain between the Ensemble and the SAXS models also parallel their distances in PCA subspace. The backbone RMSD values of the D1-D2 domain between the models are between 2.5 and 4.3 Å comparable to dynamical fluctuation. Larger RMSD values of the D1-D3 domain (see Table 1) are attributed to the twisted D3 domain relative to the D1-D2 domain. A small twisted angle between domains tends to give a larger RMSD value. Furthermore, aMD sampled additional conformational spaces presumably lower populated ST2 conformations not characterized by either the crystal structures or SAXS derived models.

**Table 1:** Euclidean distances between N1-N9, M1-M8 and the SAXS derived model conformations in the PC subspace. The distance in PCA subspace is calculated using the coefficients of the PC vectors of each conformation projected to the PC subspace and is in arbitrary units (a.u.). RMSD calculations are based on the Cα, C and N atoms of the protein backbone. The states shown in bold italic font are close to nearby SAXS derived models with distances of less than 100.

Ensemble (20 ns)	N1	*N2*	N3	N4	N5	*N6*	*N7*	*N8*	*N9*
**SAXS models**	3p68	***5p93***	9p31	7p26	7p26	***9p31***	***7p22***	***3p68***	***5p96***
**Distance (a.u.)**	320	***98***	162	174	117	***63***	***53***	***76***	***89***
**RMSD (Å): D1-D3**	18	***10***	15	8	8	***7***	***8***	***12***	***10***
**RMSD (Å): D1-D2**	3.8	***2*.*5***	3.4	2.7	2.7	***2*.*9***	***3*.*1***	***2*.*7***	***3***
**Ensemble (30 ns)**	M1	***M2***	***M3***	***M4***	M5	M6	***M7***	M8	
**SAXS models**	3p68	***3p68***	***5p93***	***7p26***	9p31	7p26	***7p22***	3p68	
**Distance (a.u.)**	317	***94***	***92***	***105***	165	170	***50***	372	
**RMSD (Å): D1-D3**	18	***13***	***10***	***7***	15	8	***7***	13	
**RMSD (Å): D1-D2**	4.3	***3*.*2***	***2*.*5***	***3*.*4***	2.8	3	***3*.*4***	2.8	

### The conformational ensemble of ST2^ECD^ and their binding with IL-33

**Fig 5:**
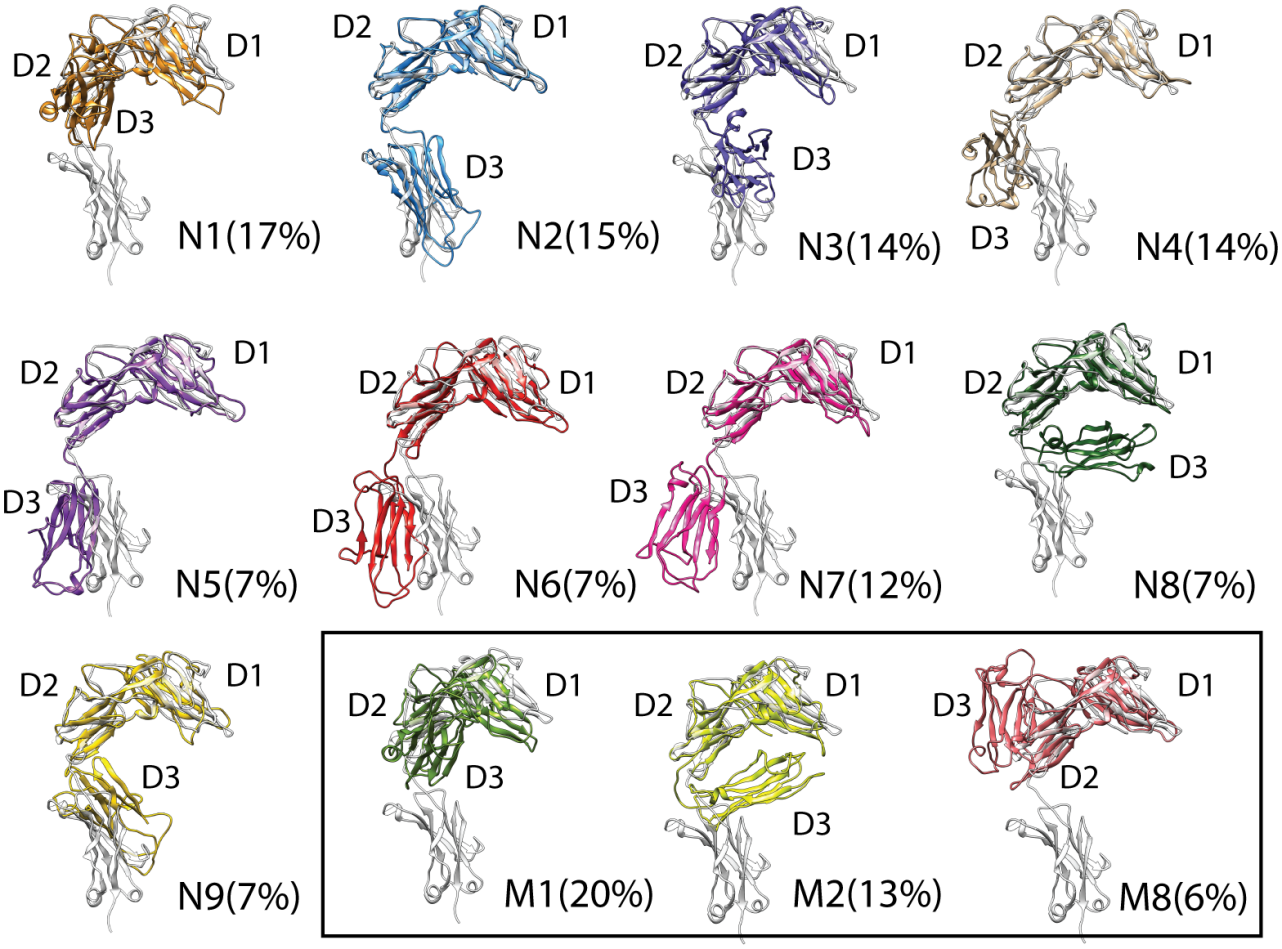
Conformations closest to the centroids of the cluster groups from mean shift cluster analysis. Each conformation was aligned to the IL-33 bound ST2 structure (grey color). The D1, D2, D3 domains of each conformation are labeled.

Alignment of ST2^ECD^ conformations corresponding to **N1**-**N9, M1, M2,** and **M8** with the IL-33 bound ST2 crystal structure are shown in Fig 5. The comparison further reaffirmed that the flexibility of ST2^ECD^ is attributed to the relative motion of the D3 domain to the D1-D2 domain. Three conformations (**M1**, **M2**, **M8**) corresponding to the trapped regions in the extended simulations from 80 to 120 ns (Fig 4A) are also provided. Of note, the D3 domain in **N1** and **M2** are in opposite orientation relative to the D1-D2 domain. Although **N8**, **N9** and **M8** are close to each other in the PC subspace, the D3 domain exhibits substantial differences in orientation. This partly reflects the ruggedness of the energy landscape in this location of the PC subspace.

**Fig 6:**
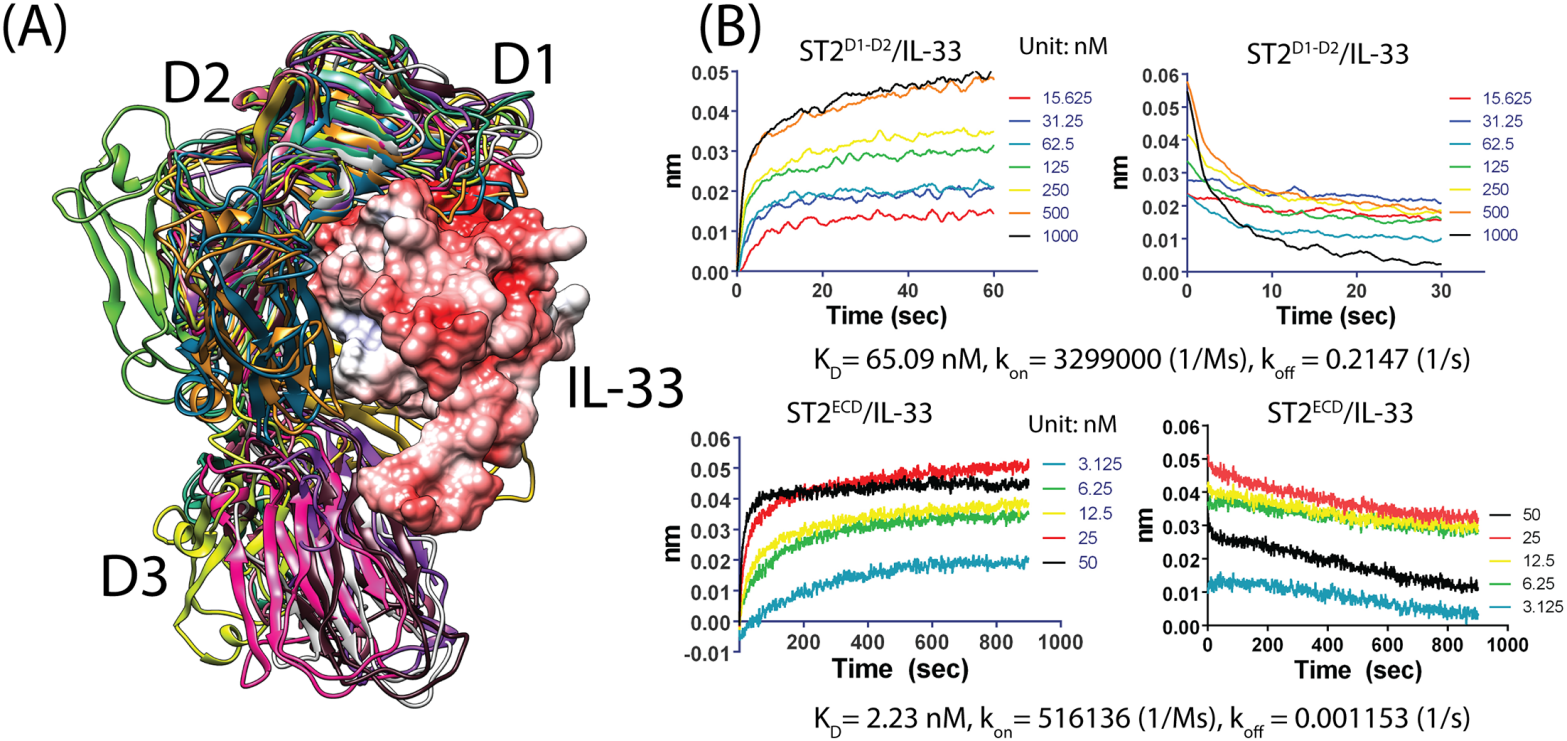
Alignment of the 12 representative conformations with ST2/IL-33 and the binding data of ST2 with IL-33. (A) The D3 domains of the 11 conformations exhibit few clashes with IL-33 after alignment with the ST2/IL-33 structure. IL-33 is shown in surface representation and colored according to the electrostatic charge map where red and white color correspond to negative and neutral charged regions. (B) Binding data between ST2^D1-D2^, ST2^ECD^ and IL-33 using Octet RED biolayer interferometry. Concentrations of IL-33 in nM used in the experiment, K_D_, k_on_ and k_off_ are provided.

Fig 5 gives a depiction of the conformational ensemble of ST2^ECD^ and theoretical pre-existing conformations without ligand binding. Visualization of these conformations inspired us to investigate if some of them may bind with IL-33. Alignment of the D1-D2 domain of **N1**-**N9** and **M1**-**M8** to the ST2^ECD^/IL-33 crystal structure showed that the D3 domain of 11 models (**N1**, **N4**, **N5**, **N6**, **N7, N9, M1, M4, M6, M7,** and **M8**) exhibited minimal clashes with IL-33 from the ST2^ECD^/IL-33 structure (see Fig 6A). The observation led us to study the contribution of the D1-D2 domain to the binding between ST2^ECD^ and IL-33. Using the Bio-Layer Interferometry (BLI)-based assay, we measured the binding affinities of IL-33 with ST2 containing the D1-D3 domain (sequence: 19–323, ST2^ECD^) and the D1-D2 domain only (sequence: 19–206, ST2^D1-D2^). We determined that the K_D_ values of ST2^ECD^/IL-33 and ST2^D1-D2^/IL-33 are 2.23 and 65.09 nM corresponding to binding free energies of -11.79 and -9.80 kcal/mol respectively. Our K_D_ value between ST2^ECD^ and IL-33 is comparable to 5 nM and 0.5–0.8 nM previously determined by the isothermal titration calorimetry assay[23] and the Biacore assay[21,23]. Based on our data, the D1-D2 domain of ST2^ECD^ contributed 83% to the binding free energy between ST2^ECD^ and IL-33. We also observed 6 versus 186 fold differences in k_on_ and k_off_ rate between ST2^ECD^, ST2^D1-D2^ and IL-33 (ST2^ECD^: k_on_ = 5.16*10^5^ 1/Ms, k_off_ = 0.001153 1/s, ST2^D1-D2^: k_on_ = 3.299*10^6^ 1/Ms, k_off_ = 0.2147 1/s (see Fig 6B). The much closer association rates between ST2^ECD^, ST2^D1-D2^ and IL-33 support the hypothesis that relatively abundant ST2^ECD^ conformations in thermodynamic equilibrium can bind effectively with IL-33 via the D1-D2 domain initially. The adaptation of the D3 domain to IL-33 follows to achieve a high affinity between ST2^ECD^ and IL-33.

### Binding sites analysis of the representative conformations of ST2^ECD^ using Sitemap

**Fig 7:**
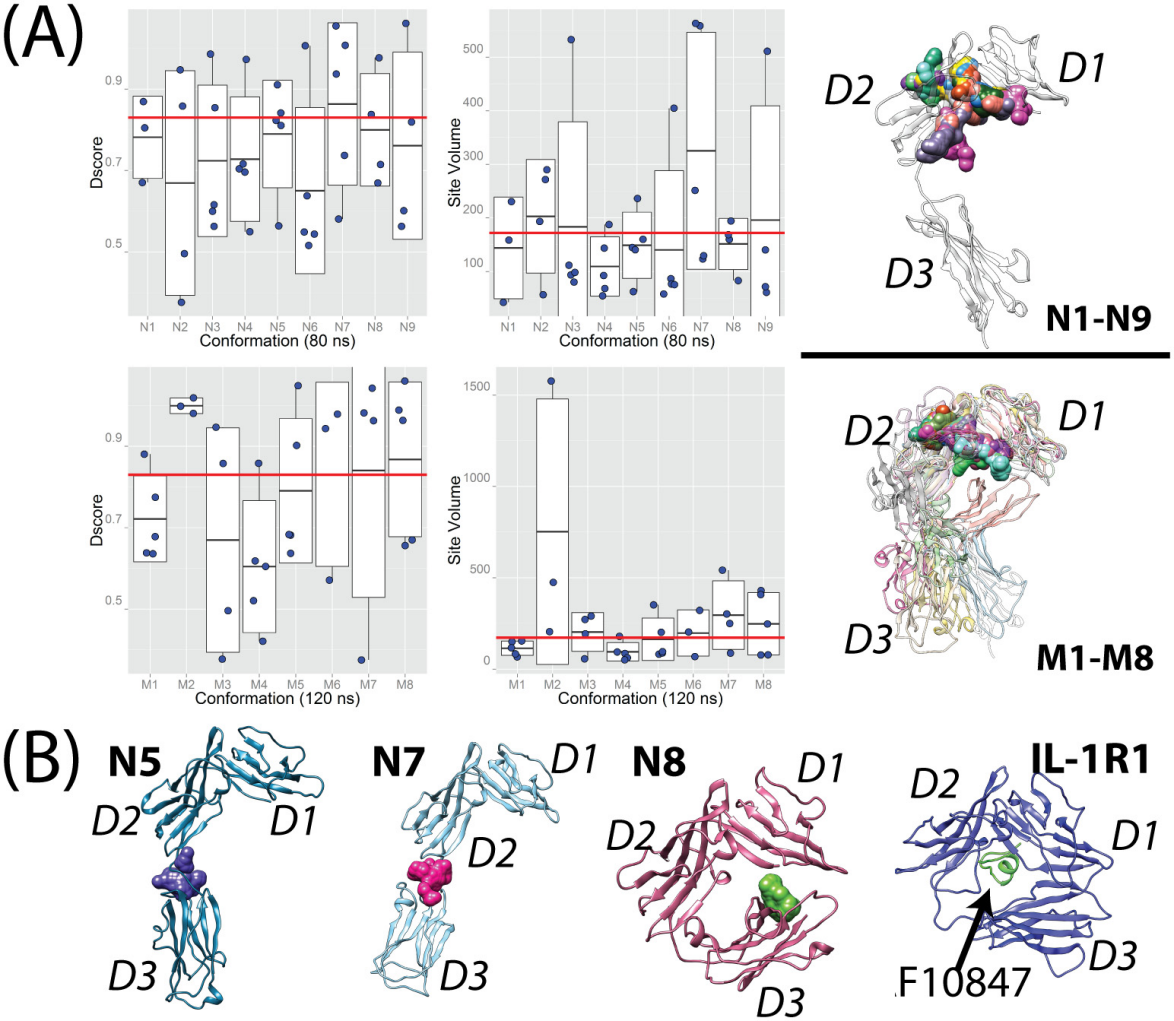
Binding sites evaluation of the representative conformations of ST2^ECD^. (A) Dscore values and Site volumes detected in **N1**-**N9** and **M1**-**M8** where the red lines represent Dscore = 0.83 (the druggable value) and Site Volume = 172 Å^3^ (the average value for the sites in the IL-33 bound ST2^ECD^ crystal structure) respectively. Druggable sites detected at the interface between the D1 and D2 domains are shown as surface shapes. The upper structure shows the druggable sites found in **N1**-**N9** using the IL-33 bound ST2 structure as a reference, and the lower one shows those found in **M1**-**M8** where all conformations were aligned. (B) Locations of druggable sites at the D2-D3 interface identified in **N5** and **N7** are colored as blue and pink. A unique site (green) between the D1 and D3 domains found in **N8** is shown and compared with the AF10847-bound IL-1R1 crystal structure.

Structures of the representative conformations permit identification and assessment of small molecule binding sites in ST2^ECD^. For this analysis, we used the Sitemap[56] program. Sitemap has been used to identify potential allosteric modulator sites in IL-1R1 in our previously report[24], in the druggability analysis of Bromodomain proteins[57], and in the protein-protein interaction sites evaluation[58]. Here, we examined two indexes from the Sitemap analysis, i.e. Dscore (defined below) and the volume of the binding sites (Site Volume) as shown in Fig 7A. The Dscore value is an overall assessment of the likelihood that the identified binding site can interact with small molecules. Previous evaluation of Sitemap on a set of co-crystal structures with known inhibitor affinities suggested that a binding site assessed with a Dscore ≥ 0.83 is "druggable"[56]. Although this measure does not account for the adaptability of the binding site to ligand binding, it prioritizes binding sites for follow-up investigation. Using this value, we found that 15 of 41 sites in **N1**-**N9** and 18 of 34 sites in **M1**-**M8** were classified as druggable sites (see Fig 7A) corresponding to 37% and 53% of all detected sites respectively. Greater than 50% of these druggable sites have site volumes less than 300 Å^3^. Nine druggable sites have volumes between 400 and 550 Å^3^. Three druggable sites detected in M2 have much higher Dscore values and larger site volumes. This is attributed to that M2 adopts a conformation similar to the AF10847-bound IL-1R1 structure in which D3 is in close contact with D1. Because **N1**-**N9** and **M1**-**M8** are representative ST2^ECD^ conformations from the conformations obtained from the aMD simulations, we expect that at least a third of detected small molecule binding sites are druggable in the conformational ensemble of ST2^ECD^.

Analysis of the druggable binding sites revealed two common locations in ST2^ECD^. Majority of the druggable sites are located at the interface between the D1 and D2 domains for all conformations analyzed as shown in Fig 7A. Two conformations, **N5** and **N7**, in which the D3 domain adopted a stretched orientation revealed a potential druggable site at the interface between the D2 and D3 domains (Fig 7B). Besides the two common locations, the D1 domain is close to the D3 domain in **N8** and formed a binding site suitable for small molecule binding (green envelop shape in Fig 7B). Conformation of **N8** is similar to the AF10847-bound IL-1R1 structure and the location of the druggable site between the D1 and D3 domains in **N8** resembles that identified in IL-1R1[24]. Whether antagonistic peptides analogous to AF10847 or small molecules can be found to bind with ST2^ECD^ adopting the **N8** conformation remains to be determined. Among these druggable sites, the sites at the interface between the D1 and D2 domains frequently gave the highest Dscore value and were also larger in size. The shape of the sites at the interface between the D2 and D3 domains however show greater variability and are dictated by the relative orientation between the D2 and D3 domains.

## Conclusion

Small-angle X-ray scattering has been widely used to investigate protein conformations in solution[59,60]. Structural models constructed by fitting to the SAXS data gave additional information of the protein conformations in solution to corroborate with crystal structures or NMR data. They can be particularly valuable to provide architectures of protein conformations in solution when NMR structures are unavailable for large proteins or protein complexes. In this study, we incorporated the backbone conformations from the SAXS-derived models (ligand-free) with crystal structures (ligand-bound) in the PCA to construct the conformational space of ST2^ECD^ for analyzing the conformations obtained from MD simulations. Four sets of parameters were used in the aMD simulations to efficiently sample the ST2^ECD^ conformations starting with the IL-33 bound ST2^ECD^ structure. Examination of the conformations projected to the first three principal components confirmed the extensive coverage of ST2^ECD^ conformational space from simulations. Application of the mean shift cluster analysis to all conformations identified cluster groups to represent the conformational ensemble of ST2^ECD^. Comparison of conformations at the centroid of each cluster group with ST2/IL-33 structure depicted the high mobility between the D1-D2 and D3 domains.

Alignment of ST2/IL-33 structure with the representative conformations further suggested that D3 of ST2^ECD^ in solution may not interfere with the initial binding between IL-33 and the D1-D2 domain. Experimental binding affinity measurement demonstrated that the D1-D2 domain of ST2^ECD^ contributes 83% of binding free energy between ST2^ECD^ and IL-33. We proposed a model of the binding event in which the D1-D2 domain of ST2^ECD^ initially recognizes IL-33 followed by the adaptation of the D3 domain mediated by the loop between the D2 and D3 domains. IL-1R1, another member of IL-1R family, also has the D1-D3 domains, and previous studies reported that the binding affinities (K_D_) of the D1-D2 domain of IL-1R1 to IL-1α, IL-1β and IL-1Ra are 7 μM, > 10 μM and 28 nM respectively[28] whereas the K_D_ of the D1-D3 domain of IL-1R1 with IL-1β is 2 nM[29]. IL-1Ra was further shown to interact primarily with the D1-D2 domain of IL-1R1 in a crystal structure[28]. The D1-D2 domain of IL-1R1 contributed only 58% in binding free energy with its endogenous activating cytokine IL-1β much less than that between ST2^ECD^ and IL-33. This cannot be attributed to the inability of the D1-D2 domain of IL-1R1 to potently bind with cytokines as it binds with antagonistic IL-1Ra using primarily the D1-D2 domain at a K_D_ of 28 nM. There appears to be functional differences in the cytokine signaling and regulation between IL-1R1/IL-1β and ST2/IL-33. Our hypothesis of recognition between ST2^ECD^ and IL-33 does not preclude that the membrane-bound ST2 binds to IL-33 using a different mechanism. The D3 domain of the membrane-bound ST2 is restrained at the cell membrane and can potentially modulate inter-domain motion between the D2 and D3 domains. Differences in the IL-33 recognition between membrane-bound ST2 and ST2^ECD^ remain to be determined.

Our binding site identification and assessment determined two major locations in ST2^ECD^ that are suitable for small molecule binding. Druggability evaluation informed that a third of all detected binding sites in the representative conformations have Dscore values ≥ 0.83 implying druggability. The primary and most frequently detected location (Site 1) is at the interface between the D1 and D2 domains whereas the second location (Site 2) is between the D2 and D3 domains. Site 1 yields a higher Dscore value and adopts a relatively rigid conformation as observed in simulations. Our binding data also show that the D1-D2 domain of ST2^ECD^ interacts with IL-33 potently. Compounds bound to this site can impact the inter-domain motion between D1 and D2 and interfere directly with IL-33 binding. Thus, Site 1 is an attractive location to seek small molecule inhibitors with high potencies. Higher variability in the binding site conformations was found at Site 2 because it engages the flexible D3 domain. Ligands bound to Site 2 can potentially trap the orientation of the D3 domain and cause ineffective binding between ST2^ECD^ and IL-33 similar to the strategy suggested in our study of IL-1R1[24]. Another unique site was found in **N8** when the D1 and D3 domains make close contacts. Small molecules binding to this site in **N8** can potentially stabilize **N8** similar to the antagonistic action of the AF10847 peptide imposed on IL-1R1[30,61]. Given the interest to selectively inhibit sST2 or membrane-bound ST2 to attenuate T_H_2 response, binding sites besides Site 1 need to be explored. Although we only showed representative conformations from the cluster groups in this work, other conformations in each cluster group can be exploited in *in silico* hit discovery. Computational simulations have been instrumental to facilitate the discovery of transient and cryptic pockets in proteins permissible for ligand binding in mutant p53[62], TEM-1 β-latamase[63], IL-2, Rnase H[64] and Bcl-xL[65] employing different approaches. These transient protein conformations are difficult to identify by experimental means alone. In this work, we reported another example of discovering potential small molecule binding sites in ST2 exhibiting large interdomain motion. Follow-up studies will be reported in due course.

ST2/IL-33 signaling is involved with various T-cell mediated immune responses and promotes the production of the T_H_2 associated cytokines. The ST2/IL-33 axis has been associated with pathological diseases including asthma, rheumatoid arthritis, atherosclerosis and GVHD[9,13,15,66]. Therapeutic interventions in the ST2/IL-33 axis will provide novel treatment options to these immunity-related diseases or disorders. Although antibodies are widely used to block the extensive interactions between cytokine receptors and cytokines[67], small molecule inhibitors/modulators targeting membrane-bound receptors including TLR-8[68] and FGFR[69] have been reported. In summary, our study characterized ST2^ECD^ conformations and assessed their small molecule binding sites that can be useful to discover small molecules targeting ST2^ECD^. The approach could potentially be applied to other cytokine receptors exhibiting similar domain architectures and inter-domain flexibility.

## Supporting Information

S1 Fig The Porod-Debye plot of the ST2 SAXS data. The curve continues to increase without reaching plateau at high q values (not shown).
(TIF)
